# General Pathway for a Convenient One-Pot Synthesis of Trifluoromethyl-Containing 2-amino-7-alkyl(aryl/heteroaryl)-1,8-naphthyridines and Fused Cycloalkane Analogues

**DOI:** 10.3390/molecules16042817

**Published:** 2011-03-30

**Authors:** Helio G. Bonacorso, Rosália Andrighetto, Nícolas Krüger, Nilo Zanatta, Marcos A. P. Martins

**Affiliations:** Núcleo de Química de Heterociclos (NUQUIMHE), Departamento de Química, Universidade Federal de Santa Maria, 97105-900 Santa Maria, RS, Brazil

**Keywords:** trifluoromethylated heterocyles, quinolines, naphthyridines, cycloalka[*b*][1,8]-naphthyridines, aminonaphthyridines

## Abstract

A convenient and general method for the synthesis in 26–73% yields of a new series of 7-alkyl(aryl/heteroaryl)-2-amino-5-trifluoromethyl-1,8-naphthyridines from direct cyclocondensation reactions of 4-alkoxy-1,1,1-trifluoroalk-3-en-2-ones [CF_3_C(O)CH=C(R^1^)OR, where R^1 ^= H, Me, Ph, 4-MePh, 4-OMePh, 4-FPh, 4-BrPh, 4-NO_2_Ph, 2-furyl, 2-thienyl and R = Me, Et] with 2,6-diaminopyridine (2,6-DAP), under mild conditions, is described. Another synthetic route also allowed the synthesis of 2-amino-5-trifluoromethyl-cycloalka[*b*][1,8]naphthyridines in 33–36% yields, from direct or indirect cyclo-condensation reactions of five-, six- and seven-membered 2-trifluoroacetyl-1-methoxy-cycloalkenes with 2,6-DAP.

## 1. Introduction

Among the nitrogenous heterocycles, naphthyridines and their derivatives represent an important class of organic molecules that attract the interest of both synthetic and medicinal chemists due to their exceptionally broad spectrum of biological activities as well as their use as important binding units in the molecular design of synthetic receptors [1]. Naphthyridine derivatives have attracted considerable attention primarily due to the presence of a 1,8-naphthyridine skeleton in many compounds which have been isolated from natural substances and exhibit various biological activities [2]. As a heterocyclic moiety, 1,8-naphthyridine also deserves special interest as in its molecule, the arrangement of the nitrogen atoms is optimal for chelation of various metal cations, including lanthanide ions [3].

In parallel to the growing interest in the synthesis of 1,8-naphthyridines to provide biologically active molecules, a large number of publications have reported that several of their derivatives possess antibacterial [4], antimycobacterial [5], antitumor [6], anti-inflammatory [7,8], analgesic [8], antiplatelet [9], gastric antisecretary [10], local anaesthetic [11], anticonvulsant [12] and antihypertensive activity [13,14], besides being associated with β-adrenergic blocking properties [15]. Some 1,8-naphthyridine compounds have been patented as fungicides, bactericides, insecticides, herbicides, anxiolytic, antihypertensives, antiarrhythmics and also as immunostimulants [2,16,17,18,19].

In addition, has been recognized that attachment of a trifluoromethyl group into heterocycles can be used to modulate the physical, chemical and biological properties. It is well documented that the influence of the trifluoromethyl substituent on physiological activity is due mainly to the increased lipophilicity of the molecules, causing greater cell permeability and resistance to enzyme degradation [20]. Consequently, synthetic methodology to incorporate fluorine and fluorous synthons must be improved in order to prepare sophisticated fluoroorganic molecules on a practical scale. One of the most satisfactory methods for introducing a CF_3_ group into heterocycles is via the trifluoromethylated building block approach. The trifluoroacetylation of enol ethers or acetals provided, in one step and in good yields, β-alkoxyvinyl triﬂuoromethyl ketones **1** which proved to be useful building blocks for the syntheses of many series of heterocyclic compounds [21].

Since the 50s various diamino-ketoester condensations involving reactions of cyclic and acyclic β-ketoesters or diketones with aminopyridines or diaminopyridines have been studied in an attempt to develop generalized predictions regarding the direction of ring closure to form diazepinones, naphthyridones, naphthyridines or pyrimidines [6,12,13,14,15,19,22]. Whereas a literature review shows that the synthesis of trifluoromethylated naphthyridines and derivatives has been little explored and that 1,8-naphthyridines trifluoromethylated described are associated with satisfactory biological activities [14], the incorporation of trifluoromethyl group in a variety of 1,8-naphthyridines would be expected to provide highly desirable intermediates for the synthesis of new drug candidates. So, due to the great biological importance and employment of amino-naphthyridines as starting material for the synthesis of new tri and tetracyclic heterocycles, the development of new synthetic approaches remains an active research area [19,23]. 

The use of diethyl ethoxymethylenemalonate (EMME) [24], Conrad-Limpach [25], Knorr [26] and Skraup [27] methods have been particularly successful in the synthesis of certain quinolines. The adaptation of these reactions to the synthesis of the corresponding naphthyridines by employing aminopyridines instead of anilines should furnish convenient methods for the preparation of these types of compounds since aminopyridines are readily available [19]. However, these methods have not been as satisfactory for the preparation of 1,8-naphthyridines as they are the preparation of quinolines. In contrast to aniline derivatives, 2-aminopyridine derivatives may cyclize in two ways, one of which leads to the formation of 1,8-naphthyridines and the other leads to the formation of pyrimidines, and the latter course of reaction has is observed with more frequency [28]. In both types of cyclization, the pyridine ring functions as the electron donor and the carbonyl group in the side chain serves as the electron acceptor. 

The formation of pyrimidines should not be surprising, since the resonance structures existing in the structure of the 2-aminopyridine derivatives strongly favor cyclization leading to the pyrimidine ring. Nevertheless, although the formation of a pyrimidine often occurs, an investigation of the synthesis of certain 1,8-naphthyridines from aminopyridines with diethylmalonate, ethoxymethylidenemalonate or ethyl acetoacetate has been made [14,19,29]. It is known that the synthesis of 1,8-naphthyridines has been performed successfully when 6-methyl-2-aminopyridine or 2,6-diaminopyridine are used as precursors, since 6-methyl or 6-amino groups activate the 3-position leading to those molecules [14,29]. Thus, the great difference in behavior of 2-aminopyridine and 2,6-diaminopyridine, for example, has usually been attributed to activation of the 3-position by the electron releasing amine group.

Recently, we reported reactivity of the endocyclic nitrogen atom of the π-deficient pyridine ring towards the carbonyl group of the trichloroacetyl enamine derivates from the reactions employing 4-alkoxy-4-alkyl(aryl)-1,1,1-trichloroalk-3-en-2-ones and 2-aminopyridine in a molar ratio of 1:1, presenting a convenient method to obtain 4-oxo-4*H*-pyrido[1,2-*a*]pyrimidines in good yields (45–81%) [28]. We also reported the synthesis of 5*H*-thiazolo[3,2-*a*]pyrimidi-5-ones from the reactions of β-alkoxyvinyl trichloromethyl ketones and 2-aminothiazole [30]. Unfortunately, reactions using β-alkoxyvinyl trifluoromethyl ketones and 2-aminopyridine in an attempt to obtain the respective cyclic structure only resulted in the isolation of trifluoroacetylenamine derivatives [32]. On the other hand, reactions using β-alkoxyvinyl trifluoro(chloro)methyl ketones and 2,3-diaminopyridine have been successfully employed in the synthesis of 3*H*-pyrido[2,3-*b*][1,4]diazepinols [31,32] or diazepinones [31,33].

**Scheme 1:**
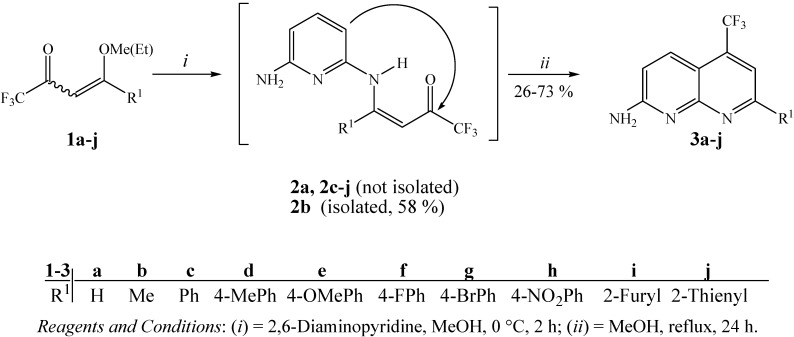
Synthesis of 7-alkyl(aryl/heteroaryl)-2-amino-5-trifluoromethyl-1,8-naphthyridines.

Although the reactions of 4-alkoxy-4-alkyl(aryl/heteroaryl)-1,1,1-trifluoroalk-3-en-2-ones with primary and secondary amines have been well documented [28,30,31,32,33,34,35,36,37,38,39,40,41], there are no reports in the literature dealing with β-alkoxyvinyl trifluoromethyl ketones as electrophilic precursors and 2,6-diaminopyridine (2,6-DAP) as nucleophilic precursor. Considering the importance of trifluoromethylated heterocycles, as an extension of our research the purpose of this paper is to report the results of a chemical behavior study of the reactions of 4-alkoxy-4-alkyl(aryl/heteroaryl)-1,1,1-trifluoroalk-3-en-2-ones and 2-trifluoroacetyl-1-methoxycycloalkenes **1** with 2,6-DAP, a symmetrical heteroaromatic diamine, aiming at the synthesis of new nitrogen-containing trifluoromethylated heterocycles with conventional procedures (Scheme 1). None of methods reported to date for the synthesis of naphthyridines employs the strategy adopted in this study. Our method allows the easier introduction of CF_3_ group at position 5 and of wide scope of both electron-donor and electron-withdrawing substituents at position 7 and fused cycloalkanes to the C6-C7 bond of the naphthyridines ring. Furthermore, the free amino group at position 2, in both cases, allows further important derivatizations. 

## 2. Results and Discussion

Initially, a series of ten examples of 4-alkoxy-4-alkyl(aryl/heteroaryl)-1,1,1-trifluoroalk-3-en-2-ones **1a-j**, which are readily available *CCC* synthetic blocks, were prepared from trifluoroacetylation reactions of enol ethers commercially available (for **1a-b**) or generated *in situ* from the respective acetophenone dimethyl acetal (for **1c-j**) with trifluoroacetic anhydride, respectively, in the presence of pyridine, as described in the literature [42,43,44,45].

Fortunately, we found that trifluoromethylated ketones **1a-j** when added dropwise to 2,6-DAP at a molar ratio of 1:1 in methanol as solvent at 0 °C for 2 hours and after heating under reflux for 24 hours, produced 7-alkyl(aryl/heteroaryl)-2-amino-5-trifluoromethyl-1,8-naphthyridines **3a-j**. These compounds were easily isolated from the one-step reaction mixtures in 51–73% yields, although **3a**, **3b** and **3i** were obtained in lower yields 26, 39 and 38%, respectively (Scheme 1). This is not surprising since a detailed review of the literature shows that enaminone derivatives of β-ethoxyvinyl trifluoromethyl ketone (enone **1a**) present a different chemical behavior from other enones [41], leading to heterocycles with lower yields [34] and the absence of cyclization has been reported in many papers [28,35]. The naphthyridine **3b** was previously also synthesized in a low yield (10–23%) by Eichler *et al.* from the reaction of 2,6-DAP and 1,1,1-trifluoropentane-2,4-dione [22].

As an extension of this study we also developed the synthesis of compound **3b **from the cyclocondensation reaction of enamino ketone intermediate **2b **in methanol at reflux temperature for 24 hours (Scheme 1). The isolation of **2b** was possible when the reaction of enone **1b** with 2,6-DAP was carried out in methanol as solvent at 0 °C for 2 hours. Unfortunately, the enamino ketones **2a**, **2c-j** could be not isolated as pure compounds, under the same or similar reaction conditions.

The structures of **3a-j** were established on the basis of ^1^H- and ^13^C-NMR spectroscopy and literature data for similar compounds (chemical shifts and spin ± spin coupling constants) [5,9,12,14,22,41,46]. According to the literature, it is well known that the proton in the 4-position of the naphthyridine nucleus shows long-range coupling with fluorine atoms of the 5-trifluoromethyl substituent, but in some cases the outer signals of the quartets can appear as shoulders on the inner signals instead of as clearly resolved quartets [22]. This splitting of the H-4 signal is seen in all of the compounds having this structural feature and was clearly seen in ^1^H-NMR spectral data of compounds **3a-j**.

**Scheme 2:**
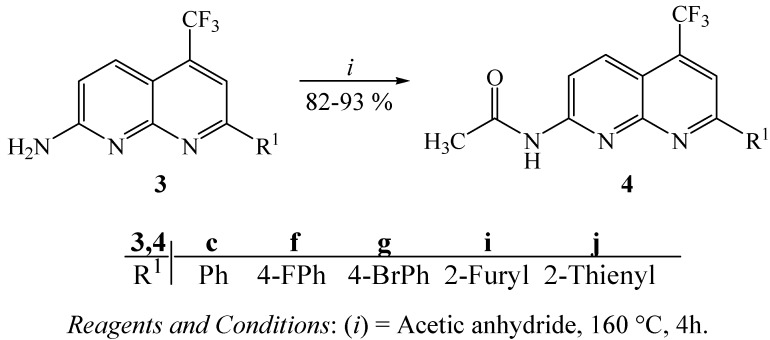
Synthesis of 2-acetamide derivatives.

Furthermore, some examples of compounds **3 **were converted in good yields (82–93%) to the corresponding 2-acetamide derivatives **4**, by reaction with acetic anhydride under high temperature [14] (Scheme 2). 

**Scheme 3:**
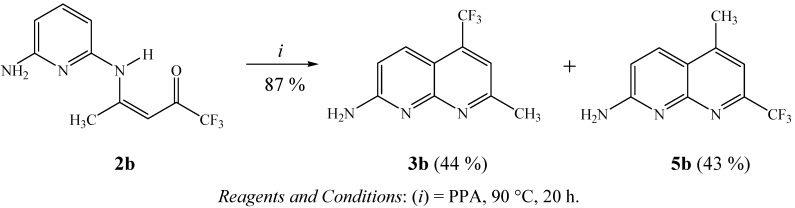
Synthesis of isomers **3b**:**5b**.

As a second extension, applying a similar cyclization method to that previously described by us for the preparation of some trifluoromethyl-substituted benzo[*h*]quinolines [35], dihydrobenzo[*c*]acridines [36], cycloalka[*b*]quinolines [37], 1,2,3,4-tetrahydroacridines [38], 7-aminoquinolines and 1,7**-**phenanthrolines [41], to investigate the chemical behavior of enamino ketone intermediate **2b** in polyphosphoric acid medium (PPA), we found that a 1:1 mixture of isomers **3b**:**5b** is obtained when **2b** is heated at 90 °C for 20 h (Scheme 3). These compounds were easily identified since CH_3_ and CF_3_ groups have different chemical shifts in the ^13^C-NMR spectra of each of the isomers. Thus, 7-methyl-2-amino-5-trifluoromethyl-1,8-naphthyridine (**3b**) showed chemical shifts at 133.8 ppm (C5, q, ^2^*J_CF _*31 Hz) and 24.6 ppm for the CH_3 _group, while the isomer 5-methyl-2-amino-7-trifluoromethyl-1,8-naphthyridine (**5b**) showed chemical shifts at 148.1 ppm (C7, q, ^2^*J_CF _*34 Hz) and 17.5 ppm for the CH_3 _group. 

**Scheme 4:**
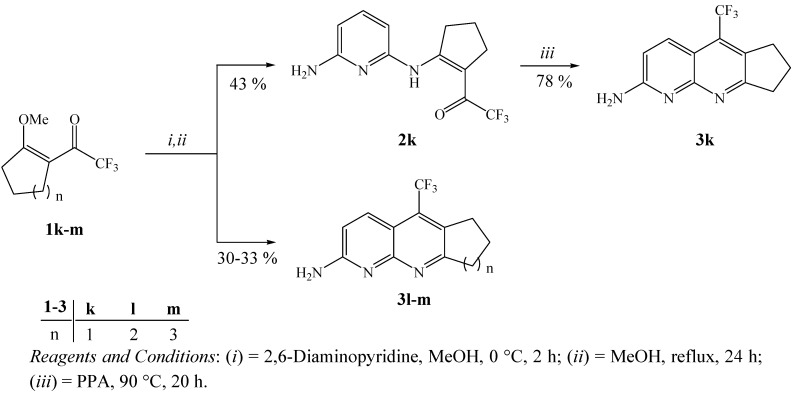
Synthesis of trifluoromethyl substituted cycloalka[*b*][1,8]naphthyridines.

As an second extension of this study, we also developed the synthesis of trifluoromethyl substituted cycloalka[*b*][1,8]naphthyridines **3** from the reactions of 2-trifluoroacetyl-1-methoxycycloalkenes **1** with 2,6-DAP (Scheme 4). Firstly, three examples of methoxycycloalkenes **1k-m** were obtained by a direct acylation reaction of the cycloalkane dimethyl acetals with trifluoracetic anhydride in the presence of pyridine, as described in the literature [47,48]. Subsequently, the intramolecular cyclization reactions of trifluoroacetylated cycloalkenes **1k-m** were carried out, applying the same conditions described for the preparation of **3a-j**. This reaction condition allows to isolate, in one-pot, only the respective cycloalka[*b*][1,8]naphthyridines **3l, 3m** in 30–33% yields because the cyclization of **1k** did not take place. This reaction condition allowed the isolation of enaminone**2k** in 43% yield, derived from 2-trifluoroacetyl-1-methoxycyclopentene. The synthesis of **3k**, in 78% yield, was only possible from intramolecular cyclization reaction of enaminone **2k** in polyphosphoric acid medium (PPA), as shown in Scheme 4. The structures of compounds** 2k, 3k-m **were easily established on the basis of ^1^H- and ^13^C-NMR spectroscopy and literature data for similar compounds, such as trifluoromethyl-containing cycloalka[*b*]quinolines [37]. 

Any attempt to explain the low yield for the synthesis of compounds **3l** and **3m** and the absence of cyclization for **1k** requires an examination of the structural effects on internal cycloalkenes **1k-m**. Information from the literature for precursors of these compounds [47], obtained by calculation using AM1 semi-empirical method, indicate the possibility of non-planar conformations for these molecules, which would explain the difficulty in achieving [3+3] intramolecular cyclization in the case of **1k** [37,48].

## 3. Experimental

### 3.1. General

Unless otherwise indicated all common reagents and solvents were used as obtained from commercial suppliers without further puriﬁcation. The melting points were determined using a Kofler Reichert-Thermovar and Electrothermal Mel-Temp 3.0 apparatus. ^1^H- and ^13^C-NMR spectra were acquired on a Bruker DPX 200 spectrometer (^1^H at 200.13 MHz) and Bruker DPX 400 (^1^H at 400.13 MHz, ^13^C at 100.32 MHz) spectrometer, 5 mm sample tubes, 298 K, digital resolution ±0.01 ppm, in DMSO-*d*_6_, in CDCl_3_ for **3a**, **3m**, **4i**, **4j** and CDCl_3_ + TFA for **4g**, using TMS as internal reference. Mass spectra were registered in a HP 6890 GC connected to a HP 5973 MSD and interfaced by a Pentium PC. The GC was equipped with a split-splitless injector, autosampler, cross-linked HP-5 capillary column (30 m, 0.32 mm of internal diameter), and helium was used as the carrier gas. The CHN elemental analyses were performed on a Perkin-Elmer 2400 CHN elemental analyzer (São Paulo University, USP/Brazil).

### 3.2. General procedure for the synthesis of (Z)-N(5,5,5-Trifluoro-4-oxo-2-penten-2-yl)-2,6-diamino-pyridine (**2b**)

To a magnetically stirred solution of 2,6-diaminopyridine (0.54 g, 5 mmol) in methanol (25 mL), a solution of **1b **(0.84 g, 5 mmol) in methanol (25 mL) was added dropwise at 0 °C over a period of 2 h. After the end of the reaction, the solvent was evaporated under reduced pressure. Then, the crude oily product was dissolved in hot ethanol and subsequently cooled (4–8 °C, 24 h) to give the title compound **2b** (0.28 g, 58% yield) as a brown solid, m.p. 130*–*132 °C.^1^H-NMR (200 MHz, DMSO-*d*_6_): *δ* = 12.69 (s, 1H, NH), 7.40 (t, *J*= 8 Hz, 1H, H-10), 6.33 (d, *J*= 8 Hz, 1H, H-9), 6.34 (d, *J*= 8 Hz, 1H, H-11), 5.49 (s, 1H, H-3), 5.20 (s, 2H, NH_2_), 2.53 (s, 3H, CH_3_). ^13^C-NMR (100 MHz, DMSO-*d*_6_): *δ* = 175.3 (q, ^2^*J*= 31 Hz, C=O), 166.5 (C-2), 157.7 (C-8), 149.0 (C-6), 138.7 (C-10), 116.6 (q, ^1^*J* = 288 Hz, CF_3_), 104.5, 103.8 (C-9, C-11), 91.8 (C-3), 21.7 (CH_3_). GC-MS (EI, 70 eV) *m/z*: 245 (M^+^, 100), 228 (15), 176 (22), 148 (15), 69 (5%). Anal. Calcd. For C_10_H_10_F_3_N_3_O (245.08): C, 48.98; H, 4.11; N, 17.14%. Found: C, 48.82; H, 4.03; N, 17.28%.

### 3.3. General procedure for the synthesis of 2-(6-Aminopyridin-2-ylamino)1-trifluoroacetyl-cyclopent-1-ene (**2k**)

To a magnetically stirred solution of 2,6-diaminopyridine (0.22 g, 2 mmol) in methanol (20 mL), a solution of **1k **(0.38 g, 2 mmol) in methanol (20 mL) was added dropwise at 0 °C over a period of 2 h. The mixture was refluxed for an additional 24 h. After the end of the reaction, the solvent was evaporated under reduced pressure. Then, the crude oily product was dissolved in hot ethanol and subsequently cooled (4–8 °C, 24 h) to give the title compound **2k** (0.22 g, 43% yield) as a brown solid, m.p. 139*–*141 °C.^ 1^H-NMR (200 MHz, DMSO-*d*_6_): *δ* = 11.85 (s, 1H, NH), 6.03 (s, 2H, NH_2_); Py: 7.39 (t, *J*= 8 Hz, 1H, H-4), 6.32 (d, *J*= 8 Hz, 1H, H-5), 6.27 (d, *J*= 8 Hz, 1H, H-3); *c*-Pent: 3.28*–*3.24 (m, 2H, CH_2_), 2.67*–*2.64 (m, 2H, CH_2_), 1.96*–*1.88 (m, 2H, CH_2_). ^13^C-NMR (100 MHz, DMSO-*d*_6_):* δ* = 172.5 (q, ^2^*J*= 33 Hz, C=O), 171.3 (C-2), 101.0 (C-1), 117.2 (q, ^1^*J*= 289 Hz, CF_3_); Py: 158.8 (C-6), 149.6 (C-2), 139.0 (C-4), 104.1, 103.5 (C-3, C-5); *c*-Pent: 34.1 (CH_2_), 26.3 (q, ^4^*J*= 3 Hz, CH_2_), 22.1 (CH_2_). GC-MS (EI, 70 eV) *m/z*: 271 (50), 256 (5), 202 (25), 174 (M^+^, 100%). Anal. Calcd. For C_12_H_12_F_3_N_3_O (271.09): C, 53.14; H, 4.46; N, 15.49%. Found: C, 53.02; H, 4.38; N, 15.61%.

### 3.4. General procedure for the synthesis of 7-alkyl(aryl/heteroaryl)-2-amino-5-trifluoromethyl-1,8-naphthyridines **3a-j**

To a magnetically stirred solution of 2,6-diaminopyridine (1.08 g, 10 mmol) in methanol (40 mL), a solution of **1a-j **(10 mmol) in methanol (40 mL) was added dropwise at 0 °C over a period of 2 h. The mixture was refluxed for an additional 24 h. After the end of the reaction, the solvent was evaporated under reduced pressure. The crude product was dissolved in ethanol and cooled (4–8 °C, 24 h). The solids **3b-j** were isolated from the cooled solution by filtration under reduced pressure. The compound **3a** was purified by flash chromatography eluting with ethyl acetate/*n*-hexane (1:2); yields: 26*–*73%. 

*2-Amino-5-trifluoromethyl-1,8-naphthyridine* (**3a**): Brown solid; yield 26%; m.p. 126*–*128 °C.^ 1^H-NMR(200 MHz, CDCl_3_): *δ* = 8.91 (d, *J*= 5 Hz, 1H, H-7), 8.08 (dq, *J*_1_= 2, *J*_2_*=* 9 Hz, 1H, H-4), 7.41 (d, *J*= 5 Hz, 1H, H-6), 6.94 (d, *J*= 9 Hz, 1H, H-3), 6.43 (s, 2H, NH_2_). ^13^C-NMR (100 MHz, CDCl_3_): *δ* = 159.9 (C-7), 156.8 (C-8a), 152.0 (C-2), 135.1 (q, ^2^*J*= 31 Hz, C-5), 134.2 (C-4), 123.1 (q, ^1^*J*= 275 Hz, CF_3_), 114.8 (C-3), 114.5 (q, ^3^*J*= 5 Hz, C-6), 112.5 (C-4a). GC-MS (EI, 70 eV) *m/z*: 214 (M^+^, 100), 194 (52), 69 (10%). Anal. Calcd. For C_9_H_6_F_3_N_3 _(213.05): C, 50.71; H, 2.84; N, 19.71%. Found: C, 50.77; H, 2.92; N, 19.81%. 

*2-Amino-5-trifluoromethyl-7-methyl-1,8-naphthyridine* (**3b**): Yellow solid; yield 39%; m.p. 200*–*202 °C (lit. [22] 195*–*197 °C).^ 1^H-NMR(200 MHz, DMSO-*d*_6_): *δ* = 8.01 (dq, *J*_1_ = 2, *J*_2_*=* 9 Hz, 1H, H-4), 7.43 (s, 1H, H-6), 7.16 (s, 2H, NH_2_), 6.97 (d, *J* = 9 Hz, 1H, H-3), 2.64 (s, 3H, CH_3_). ^13^C-NMR (100 MHz, DMSO-*d*_6_): *δ* = 160.8 (C-7), 160.7 (C-8a), 157.0 (C-2), 132.9 (q, ^2^*J =* 31 Hz, C-5), 132.4 (C-4), 123.5 (q, ^1^*J* = 275 Hz, CF_3_), 114.1 (C-3), 113.6 (q, ^3^*J* = 5 Hz, C-6), 108.8 (C-4a), 24.8 (CH_3_). GC-MS (EI, 70 eV) *m/z* 227 (M^+^, 100), 210 (3), 200 (37), 158 (3), 131 (5%).

*2-Amino-5-trifluoromethyl-7-phenyl-1,8-naphthyridine* (**3c**): Beige solid; yield 69%; m.p. 255*–*257 °C. ^1^H-NMR (200 MHz, DMSO-*d*_6_): *δ* = 8.28 (dd, *J*_1_ = 2, *J*_2_ = 8 Hz, 2H, Ph), 8.06 (dq, *J*_1_ = 2, *J*_2_*=* 9 Hz, 1H, H-4), 8.03 (s, 1H, H-6), 7.58-7.53 (m, 3H, Ph), 7.18 (s, 2H, NH_2_), 7.05 (d, *J* = 9 Hz, 1H, H-3). ^13^C-NMR (100 MHz, DMSO-*d*_6_): *δ* = 161.0 (C-7), 157.5 (C-8a), 157.2 (C-2), 137.7 (C-Ph), 134.0 (q, ^2^*J* = 31 Hz, C-5), 132.3 (q, ^4^*J* = 2 Hz, C-4), 129.8, 128.5, 127.0 (5 C-Ph), 123.2 (q, ^1^*J* = 275 Hz, CF_3_), 114.8 (C-3), 110.2 (q, ^3^*J* = 5 Hz, C-6), 110.0 (q, ^3^*J* = 2 Hz, C-4a). GC-MS (EI, 70 eV) *m/z*: 290 (M^+^, 100), 270 (23%). Anal. Calcd. For C_15_H_10_F_3_N_3_ (289.08): C, 62.28; H, 3.48; N, 14.53%. Found: C, 62.12; H, 3.60; N, 14.30%.

*2-Amino-5-trifluoromethyl-7-(4-methylphenyl)-1,8-naphthyridine* (**3d**): Yellow solid; yield 51%; m.p. 219*–*221 °C. ^1^H-NMR (200 MHz, DMSO-*d*_6_): *δ* = 8.19 (d, *J* = 8 Hz, 2H, Ph), 8.09 (dd, *J*_1_ = 2, *J =* 9 Hz, 1H, H-4), 7.98 (s, 1H, H-6), 7.35 (d, *J* = 8 Hz, 2H, Ph), 7.20 (s, 2H, NH_2_), 7.08 (d, *J* = 9 Hz, 1H, H-3), 2.40 (s, 3H, CH_3_).^13^C-NMR (100 MHz, DMSO-*d*_6_): *δ* = 160.9 (C-7), 157.4 (C-8a), 157.2 (C-2), 139.5, 134.9 (2 C-Ph), 133.8 (q, ^2^*J* = 31 Hz, C-5), 132.1 (C-4), 129.1, 126.8 (4 C-Ph), 123.2 (q, ^1^*J* = 275 Hz, CF_3_), 114.5 (C-3), 109.8 (q, ^3^*J* = 5 Hz, C-6), 99.5 (C-4a), 20.5 (CH_3_). GC-MS (EI, 70 eV) *m/z*: 303 (M^+^, 100), 276 (12), 234 (14%). Anal. Calcd. For C_16_H_12_F_3_N_3_ (303.1): C, 63.36; H, 3.99; N, 13.86%. Found: C, 63.44; H, 4.06; N, 14.01%.

*2-Amino-5-trifluoromethyl-7-(4-methoxyphenyl)-1,8-naphthyridine* (**3e**): Beige solid; yield 53%; m.p. 210*–*212 °C. ^1^H-NMR (200 MHz, DMSO-*d*_6_): *δ* = 8.32 (d, *J* = 8 Hz, 2H, Ph), 8.10 (dq, *J*_1_ = 2, *J*_2_*=* 9 Hz, 1H, H-4), 8.03 (s, 1H, H-6), 7.44 (s, 2H, NH_2_), 7.13 (d, *J* = 8 Hz, 2H, Ph), 7.10 (d, *J* = 9 Hz, 1H, H-3), 3.89 (s, 3H, CH_3_). ^13^C-NMR (100 MHz, DMSO-*d*_6_): *δ* = 161.2 (C-7), 160.9 (C-8a), 157.4 (C-Ph), 157.3 (C-2), 134.0 (q, ^2^*J* = 31 Hz, C-5), 132.5 (C-4), 130.2, 128.7 (3 C-Ph), 123.4 (q, ^1^*J* = 275 Hz, CF_3_), 114.5 (C-3), 114.1 (2 C-Ph), 109.8 (q, ^3^*J* = 5 Hz, C-6), 109.6 (C-4a), 55.2 (OCH_3_). GC-MS (EI, 70 eV) *m/z*: 320 (M^+^, 100), 300 (22%). Anal. Calcd. For C_16_H_12_F_3_N_3_O (319.09): C, 60.19; H, 3.79; N, 13.16%. Found: C, 59.90; H, 3.89; N, 13.07%.

*2-Amino-5-trifluoromethyl-7-(4-fluorophenyl)-1,8-naphthyridine* (**3f**): Yellow solid; yield 73%; m.p. 260*–*262 °C. ^1^H-NMR (200 MHz, DMSO-*d*_6_): *δ* = 8.36*–*8.32 (m, 2H, FPh), 8.06 (dq, *J*_1_ = 2, *J*_2_*=* 9 Hz, 1H, H-4), 8.01 (s, 1H, H-6), 7.38*–*7.33 (m, 2H, FPh), 7.14 (s, 2H, NH_2_), 7.02 (d, *J* = 9 Hz, 1H, H-3). ^13^C- NMR (100 MHz, DMSO-*d*_6_): *δ* = 163.4 (d,^ 1^*J* = 247 Hz, C-FPh), 161.2 (C-7), 157.3 (C-8a), 156.5 (C-2), 134.2 (d,^ 4^*J* = 3 Hz, C-FPh), 134.1 (q, ^2^*J* = 31 Hz, C-5), 132.4 (C-4), 129.5 (d,^ 3^*J* = 9 Hz, 2 C-FPh), 123.3 (q, ^1^*J* = 275 Hz, CF_3_), 115.6 (d, ^2^*J* = 21 Hz, 2 C-FPh), 115.1 (C-3), 110.2 (q, ^3^*J* = 5 Hz, C-6), 110.0 (q, ^3^*J* = 2 Hz, C-4a). GC-MS (EI, 70 eV) *m/z*: 307 (M^+^, 100), 238 (25), 164 (2), 143 (4%). Anal. Calcd. For C_15_H_9_F_4_N_3_ (307.07): C, 58.64; H, 2.95; N, 13.68%. Found: C, 58.48; H, 3.11; N, 13.24%.

*2-Amino-7-(4-bromophenyl)-5-trifluoromethyl-1,8-naphthyridine* (**3g**): Yellow solid; yield 73%; m.p. 285*–*287 °C. ^1^H-NMR (200 MHz, DMSO-*d*_6_): *δ* = 8.23 (dd, *J*_1_ = 2, *J*_2_ = 8 Hz, 2H, Ph), 8.08 (dq, *J*_1_ = 2, *J*_2_*=* 9 Hz, 1H, H-4), 8.02 (s, 1H, H-6), 7.72 (dd, *J*_1_ = 2, *J*_2_ = 8 Hz, 2H, Ph), 7.18 (s, 2H, NH_2_), 7.07 (d, *J* = 9 Hz, 1H, H-3). ^13^C-NMR (100 MHz, DMSO-*d*_6_):*δ* = 161.3 (C-7), 157.3 (C-8a), 156.4 (C-2), 136.9 (C-Ph), 134.2 (q, ^2^*J* = 31 Hz, C-5), 132.5 (C-4), 131.7, 129.2, 123.9 (5 C-Ph), 123.4 (q, ^1^*J* = 275 Hz, CF_3_), 115.3 (C-3), 110.4 (C-4a), 110.2 (q, ^3^*J* = 5 Hz, C-6). GC-MS (EI, 70 eV) *m/z*: 367 (M^+^, 100), 298 (12), 288 (50), 144 (17%). Anal. Calcd. For C_15_H_9_BrF_3_N_3_ (366.99): C, 48.94; H, 2.46; N, 11.41%. Found: C, 49.04; H, 2.54; N, 11.37%.

*2-Amino-5-trifluoromethyl-7-(4-nitrophenyl)-1,8-naphthyridine* (**3h**): Yellow solid; yield 63%; m.p. > 340 °C. ^1^H-NMR (200 MHz, DMSO-*d*_6_): *δ* = 8.53 (d, *J* = 8 Hz, 2H, Ph), 8.35 (d, *J* = 8 Hz, 2H, Ph), 8.06 (dd, *J*_1_ = 2, *J =* 9 Hz, 1H, H-4), 8.02 (s, 1H, H-6), 7.20 (s, 2H, NH_2_), 7.06 (d, *J* = 9 Hz, 1H, H-3).^ 13^C-NMR (100 MHz, DMSO-*d*_6_): *δ* = 161.1 (C-7), 157.1 (C-8a), 155.0 (C-2), 148.0, 143.4 (2 C-Ph), 134.3 (q, ^2^*J* = 31 Hz, C-5), 132.1 (C-4), 128.2, 123.5 (4 C-Ph), 123.0 (q, ^1^*J* = 275 Hz, CF_3_), 115.7 (C-3), 110.9 (C-4a), 109.8 (q, ^3^*J* = 2 Hz, C-6). GC-MS (EI, 70 eV) *m/z*: 334 (M^+^, 100), 288 (97), 144 (17%). Anal. Calcd. For C_15_H_9_F_3_N_4_O_2 _(334.07): C, 53.90; H, 2.71; N, 16.76%. Found: C, 53.85; H, 2.78; N, 16.52%.

*2-Amino-5-trifluoromethyl-7-(2-furyl)-1,8-naphthyridine* (**3i**): Yellow solid, yield 38%; m.p. 230*–*232 °C. ^1^H-NMR (200 MHz, DMSO-*d*_6_): *δ* = 7.98 (d, *J* = 4 Hz, 1H, furyl), 7.89 (dq, *J*_1_ = 2, *J*_2_ = 9 Hz, 1H, H-4), 7.80 (s, 1H, H-6), 7.40 (d, *J* = 5 Hz, 1H, furyl), 7.17 (s, 2H, NH_2_), 7.04 (d, *J* = 9 Hz, 1H, H-3), 6.75 (t, *J* = 4 Hz, 1H, furyl). ^13^C-NMR (100 MHz, DMSO-*d*_6_): *δ* = 160.9 (C-7), 157.0 (C-8a), 152.3 (C-2), 149.5, 145.1, (2 C-furyl), 133.9 (q, ^2^*J* = 31 Hz, C-5), 132.3 (q, ^4^*J* = 2 Hz, C-4), 123.0 (q, ^1^*J* = 275 Hz, CF_3_), 114.5 (C-3), 112.4, 111.1 (2 C-furyl), 109.8 (q, ^3^*J* = 2 Hz, C-4a), 108.6 (q, ^3^*J* = 5 Hz, C-6). GC-MS (EI, 70 eV) *m/z*: 279 (M^+^, 100), 251 (25), 223 (12%). Anal. Calcd. For C_13_H_8_F_3_N_3_O (279.06): C, 55.92; H, 2.89; N, 15.05%. Found: C, 55.48; H, 2.88; N, 14.72%.

*2-Amino-5-trifluoromethyl-7-(2-thienyl)-1,8-naphthyridine* (**3j**): Yellow solid; yield 60%; m.p. 260*–*262 °C.^ 1^H-NMR (200 MHz, DMSO-*d*_6_): *δ* = 8.12 (d, *J* = 4 Hz, 1H, thienyl), 8.07 (dq, *J*_1_ = 2, *J*_2_ = 9 Hz, 1H, H-4), 8.04 (s, 1H, H-6), 7.78 (d, *J* = 5 Hz, 1H, thienyl), 7.26 (t, *J* = 4 Hz, 1H, thienyl), 7.23 (s, 2H, NH_2_), 7.07 (d, *J* = 9 Hz, 1H, H-3). ^13^C-NMR (100 MHz, DMSO-*d*_6_): *δ* = 161.1 (C-7), 157.0 (C-8a), 153.2 (C-2), 143.9 (C-thienyl), 133.9 (q, ^2^*J* = 31 Hz, C-5), 132.3 (q, ^4^*J* = 2 Hz, C-4), 130.0, 128.3, 127.5 (3 C-thienyl), 123.1 (q, ^1^*J* = 275 Hz, CF_3_), 114.3 (C-3), 109.9 (q, ^3^*J* = 2 Hz, C-4a), 109.3 (q, ^3^*J* = 5 Hz, C-6). GC-MS (EI, 70 eV) *m/z*: 295 (M^+^, 100), 268 (25), 226 (4%). Anal. Calcd. For C_13_H_8_F_3_N_3_S (295.04): C, 52.88; H, 2.73; N, 14.23%. Found: C, 52.94; H, 2.85; N, 14.10%.

### 3.5. General procedure for the synthesis of 2-amino-5-trifluoromethyl-7,8-dihydro-6H-cyclopenta[b][1,8]-naphthyridine (**3k**)

To a stirred mixture of H_3_PO_4_ (0.8 mL) and P_2_O_5_ (1.2 g) (PPA) at 90 °C, **2k **(0.27 g, 1 mmol) was added. The reaction mixture was stirred for an additional 20 h. After cooling, the reaction mixture was treated with crushed ice and with concentrated NH_4_OH until the pH was 8. The compound **3k** wasisolated of the solution by filtration at reduced pressure as a brown solid, in 78% yield, m.p. 172*–*174 ºC. ^1^H-NMR (200 MHz, DMSO-*d*_6_): *δ* = 8.00 (dq, *J*_1_ = 2, *J*_2_ = 9 Hz, 1H, H-4), 6.91 (d, *J* = 9 Hz, 1H, H-3), 6.80 (s, 2H, NH_2_), 3.18*–*3.14 (m, 2H, CH_2_), 3.02 (t, *J* = 9 Hz, 2H, CH_2_), 2.15*–*2.07 (m, 2H, CH_2_). ^13^C-NMR (100 MHz, DMSO-*d*_6_): *δ* = 170.0 (C-9a), 159.8 (C-2), 156.5 (C-10a), 132.5 (q, ^4^*J* = 3 Hz, C-4), 128.0 (q, ^3^*J* = 3 Hz, C-5a), 127.6 (q, ^2^*J* = 30 Hz, C-5), 124.2 (q, ^1^*J* = 276 Hz, CF_3_), 113.0 (C-3), 109.2 (q, ^3^*J* = 2 Hz, C-4a), 33.7, 29.7 (q, ^4^*J* = 2 Hz), 21.9 (3 CH_2_). GC-MS (EI, 70 eV) *m/z*: 253 (M^+^, 100), 237 (5), 184 (20%). Anal. Calcd. For C_12_H_10_F_3_N_3_ (253.08): C, 56.92; H, 3.98; N, 16.59%. Found: C, 56.80; H, 3.84; N, 16.73%.

### 3.6. General procedure for the synthesis of 2-amino-5-trifluoromethyl-cycloalka[b][1,8]-naphthyridines **3l, 3m**

To a magnetically stirred solution of 2,6-diaminopyridine (0.22 g, 2 mmol) in methanol (20 mL), a solution of **1k-l **(2 mmol) in methanol (20 mL) was added drop wise at 0 °C over a period of 2 h. The mixture was refluxed for an additional 24 h. After the end of the reaction, the solvent was evaporated under reduced pressure. The compounds **3k-l** were purified by flash chromatography eluting with ethyl acetate/*n*-hexane (1:2); yields: 30*–*33%. 

*2-Amino-5-trifluoromethyl-6,7,8,9-tetrahydrobenzo[b][1,8]naphthyridine* (**3l**): Brown solid, yield 33%; m.p. 128*–*130 °C.^1^H-NMR (200 MHz, DMSO-*d*_6_): *δ* = 8.07 (dq, *J*_1_ = 2, *J*_2_ = 9 Hz, 1H, H-4), 6.93 (d, *J* = 9 Hz, 1H, H-3), 6.85 (s, 2H, NH_2_), 2.99*–*2.96 (m, 4H, CH_2_), 1.90*–*1.78 (m, 4H, CH_2_). ^13^C-NMR (100 MHz, DMSO-*d*_6_): *δ* = 160.9 (C-2), 159.8 (C-10a), 154.6 (C-9a), 132.7 (q, ^4^*J* = 3 Hz, C-4), 130.3 (q, ^2^*J* = 28 Hz, C-5), 124.7 (q, ^1^*J* = 278 Hz, CF_3_), 124.5 (C-5a), 114.0 (C-3), 110.5 (q, ^3^*J* = 2 Hz, C-4a), 33.8, 25.6, 22.0, 21.2 (4 CH_2_). GC-MS (EI, 70 eV) *m/z*: 268 (M^+^, 100), 248 (20), 198 (9%). Anal. Calcd. For C_13_H_12_F_3_N_3 _(267.1): C, 58.42; H, 4.53; N, 15.72%. Found: C, 58.59; H, 4.65; N, 15.61%.

*2-Amino-5-trifluoromethyl-7,8,9,10-tetrahydro-6H-cyclohepta[b][1,8]naphthyridine* (**3m**): Brown solid; yield 30%; m.p. 93*–*95 °C.^ 1^H-NMR (200 MHz, CDCl_3_): *δ* = 8.20 (d, *J* = 9 Hz, 1H, H-4), 7.75 (d, *J* = 9 Hz, 1H, H-3), 7.19 (s, 2H, NH_2_), 3.21*–*3.00 (m, 4H, CH_2_), 1.79*–*1.68 (m, 6H, CH_2_). ^13^C-NMR (100 MHz, CDCl_3_): *δ* = 158.8 (C-2), 156.5 (C-11a), 154.5 (C-10a), 140.7 (C-5a), 134.6 (C-4), 131.2 (q, ^2^*J* = 30 Hz, C-5), 123.9 (q, ^1^*J* = 278 Hz, CF_3_), 113.5 (C-3), 97.3 (C-4a), 39.0, 38.9, 29.3, 27.5, 26.2 (5 CH_2_). GC-MS (EI, 70 eV) *m/z*: 281 (M^+^, 100), 265 (15), 252 (30), 212 (4%). Anal. Calcd. For C_14_H_14_F_3_N_3_ (281.1): C, 59.78; H, 5.02; N, 14.94%. Found: C, 59.69; H, 4.88; N, 15.01%.

### 3.7. General procedure for the synthesis of 2-acetylamino-7-(aryl/heteroaryl)-5-trifluoromethyl-1,8-naphthyridines **4**

A suspension of 2 mmol of amino derivatives **3c**, **3f**,**3g**, **3i** or **3j **in 5 mL of acetic anhydride was refluxed at 160 °C for 2 h. After cooling, the solids were collected and washed with water to give their acetamide derivatives **4**, in 82–93% yields.

*2-Acetylamino-5-trifluoromethyl-7-phenyl-1,8-naphthyridine* (**4c**): Beige solid; yield 74%; m.p. 224*–*226 °C. ^1^H-NMR (200 MHz, DMSO-*d*_6_): *δ* = 11.34 (s, 1H, NH), 8.54-850 (m, 2H, H-3, H-4), 8.40 (s, 1H, H-6), 8.37-835 (m, 2H, Ph), 760-7.58 (m, 3H, Ph), 2.22 (CH_3_). ^13^C-NMR (100 MHz, DMSO-*d*_6_): *δ* = 171.6 (C=0), 170.0 (C-8a), 158.7 (C-7), 155.5 (C-2), 136.9 (C-Ph), 135.4 (q, ^2^*J* = 33 Hz, C-5), 134.4 (C-4), 130.4, 128.7, 127.3 (5 C-Ph), 122.9 (q, ^1^*J* = 275 Hz, CF_3_), 115.9 (C-3), 114.4 (q, ^3^*J* = 5 Hz, C-6), 113.1 (C-4a), 23.9 (CH_3_). GC-MS (EI, 70 eV) *m/z*: 331 (40), 288 (M+, 100), 262 (15), 219 (9%). Anal. Calcd. For C_17_H_12_F_3_N_3_O (331.09) C, 61.63; H, 3.65; N, 12.68%. Found: C, 61.49; H, 3.50; N, 12.78%.

*2-Acetylamino-5-trifluoromethyl-7-(4-fluorophenyl)-1,8-naphthyridine* (**4f**): Beige solid; yield 83%; m.p. 263*–*265 °C. ^1^H-NMR (200 MHz, CDCl_3_): *δ* = 11.21 (s, 1H, NH), 8.50 (d, *J* = 9 Hz, 1H, H-3), 8.45 (d, *J* = 9 Hz, 1H, H-4), 8.40-8.37 (m, 2H, FPh), 8.33 (s, 1H, H-6), 7.39-7.35 (m, 2H, FPh), 2.23 (s, 3H, CH_3_). ^13^C-NMR (100 MHz, DMSO-*d*_6_): *δ* = 170.0 (C=0), 163.6 (d,^ 1^*J* = 248 Hz, C-FPh), 157.6 (C-8a), 155.0 (C-7), 154.9 (C-2), 135.5 (q, ^2^*J* = 33 Hz, C-5), 134.3 (C-4), 133.4 (d,^ 4^*J* = 3 Hz, C-FPh), 129.6 (d,^ 3^*J* = 9 Hz, 2 C-FPh), 122.7 (q, ^1^*J* = 275 Hz, CF_3_), 115.8 (C-6), 115.6 (d, ^2^*J* = 21 Hz, 2 C-FPh), 114.2 (C-3), 113.0 (C-4a), 23.8 (CH_3_). GC-MS (EI, 70 eV) *m/z*: 349 (45), 307 (M^+^, 100), 280 (25), 238 (15%). Anal. Calcd. For C_17_H_11_F_4_N_3_O (349.08): C, 58.46; H, 3.17; N, 12.03%. Found: C, 58.28; H, 3.02; N, 12.15%. 

*2-Acetylamino-7-(4-bromophenyl)-5-trifluoromethyl-1,8-naphthyridine* (**4g**): Beige solid; yield 93%; m.p. 222*–*224°C. ^1^H-NMR (200 MHz, CDCl_3 _+ TFA): *δ* = 11.34 (s, 1H, NH), 8.90 (d, *J* = 9 Hz, 1H, H-3), 8.45 (s, 1H, H-6), 8.18 (d, *J* = 9 Hz, 2H, Ph), 7.80 (d, *J* = 9 Hz, 2H, Ph), 7.74 (d, *J* = 9 Hz, 1H, H-4), 2.54 (s, 3H, CH_3_). ^13^C-NMR (100 MHz, CDCl_3 _+ TFA): *δ* = 176.0 (C=0), 169.8 (C-8a), 152.1 (C-7), 145.4 (C-2), 143.0 (C-Ph), 137.7 (q, ^2^*J* = 33 Hz, C-5), 133.5 (C-4), 133.0, 129.7, 128.8, (5 C-Ph), 121.6 (q, ^1^*J* = 275 Hz, CF_3_), 118.2 (q, ^3^*J* = 5 Hz, C-6), 114.1 (C-3), 113.3 (C-4a), 24.2 (CH_3_). GC-MS (EI, 70 eV) *m/z*: 409 (42), 367 (M^+^, 100), 339 (5), 296 (6), 288 (42%). Anal. Calcd. For C_17_H_11_BrF_3_N_3_O (409.1): C, 49.78; H, 2.70; N, 10.24%. Found: C, 49.61; H, 2.58; N, 10.33%.

*2-Acetylamino-5-trifluoromethyl-7-(2-furyl)-1,8-naphthyridine* (**4i**): Yellow solid; yield 82%; m.p. 221*–*223 °C. ^1^H-NMR (200 MHz, CDCl_3_): *δ* = 9.74 (s, 1H, NH), 8.60 (d, *J* = 9 Hz, 1H, H-3), 8.41 (d, *J* = 9 Hz, 1H, H-4), 8.10 (s, 1H, H-6), 7.65 (s, 1H, furyl), 7.45 (d, *J* = 3 Hz, 1H, furyl), 6.62 (t, *J* = 2 Hz, 1H, furyl), 2.29 (s, 3H, CH_3_). ^13^C-NMR (100 MHz, CDCl_3_): *δ* = 176.2 (C=0), 169.8 (C-8a), 152.5 (C-7), 151.8 (C-2), 145.3 (2 C-furyl), 136.0 (q, ^2^*J* = 32 Hz, C-5), 135.9 (C-4), 122.8 (q, ^1^*J* = 275 Hz, CF_3_), 115.8 (C-3), 114.3 (C-4a), 113.8 (q, ^3^*J* = 5 Hz, C-6), 112.9, 112.7 (2 C-furyl), 24.5 (CH_3_). GC-MS (EI, 70 eV) *m/z*: 321 (50), 306 (5), 279 (M+, 100), 251 (10%). Anal. Calcd. For C_15_H_10_F_3_N_3_O_2_ (321.07) C, 56.08; H, 3.14; N, 13.08%. Found: C, 56.00; H, 3.03; N, 13.18%.

*2-Acetylamino-5-trifluoromethyl-7-(2-thienyl)-1,8-naphthyridine* (**4j**): Beige solid; yield 90%; m.p. 225*–*227 °C.^ 1^H-NMR (200 MHz, CDCl_3_):* δ* = 9.76 (s, 1H, NH), 8.59 (d, *J* = 9 Hz, 1H, H-3), 8.40 (d, *J* = 9 Hz, 1H, H-4), 7.98 (s, 1H, H-6), 7.84 (s, 1H, thienyl), 7.54 (d, *J* = 4 Hz, 1H, thienyl), 7.16 (d, *J* = 3 Hz, 1H, thienyl), 2.29 (s, 3H, CH_3_). ^13^C-NMR (100 MHz, CDCl_3_): *δ* = 176.1 (C=0), 169.8 (C-8a), 155.3 (C-7), 154.9 (C-2), 143.4 (C-thienyl), 135.9 (q, ^2^*J* = 33 Hz, C-5), 135.7 (C-4), 130.9, 128.4, 127.9 (3 C-thienyl), 122.9 (q, ^1^*J* = 275 Hz, CF_3_), 115.8 (C-3), 114.3 (C-4a), 114.1 (q, ^3^*J* = 5 Hz, C-6), 24.7 (CH_3_). GC-MS (EI, 70 eV) *m/z*: 337 (43), 322 (5), 295 (M+, 100), 268 (25%). Anal. Calcd. For C_15_H_10_F_3_N_3_OS (337.05): C, 53.41; H, 2.99%; N, 12.46. Found: C, 53.33; H, 2.81; N, 12.54%.

### 3.8. General procedure for the synthesis of 2-amino-5-trifluoromethyl-7-methyl-1,8-naphthyridine (3b) and 2-amino-7-trifluoromethyl-5-methyl-1,8-naphthyridine (**5b**)

These compounds were obtained as a yellow solid, a 1:1 mixture of **3b**:**5b **in 87% yield, m.p. 179*–*181 °C. To a stirred mixture of H_3_PO_4_ (0.8 mL) and P_2_O_5_ (1.2 g) (PPA) at 90 °C, **2b **(0.24 g, 1 mmol) was added. The reaction mixture was stirred for an additional 20 h. After cooling, the reaction mixture was treated with crushed ice and with concentrated NH_4_OH until the pH was 8. The solution was then extracted with ethyl acetate (3 × 20 mL), the combined extracts were dried (MgSO_4_) and evaporated to dryness under a vacuum to obtain the solid mixture of isomers **3b**:**5b**. 

*2-Amino-5-trifluoromethyl-7-methyl-1,8-naphthyridine* (**3b**):Yield 44%. ^1^H-NMR (200 MHz, CDCl_3_): *δ* = 8.08 (d, *J =* 9 Hz, 1H, H-4), 7.36 (s, 1H, H-6), 6.98 (d, *J* = 9 Hz, 1H, H-3), 6.19 (s, 2H, NH_2_), 2.74 (s, 3H, CH_3_). ^13^C-NMR (100 MHz, DMSO-*d*_6_): *δ* = 160.5 (C-7), 159.8 (C-8a), 156.5 (C-2), 133.8 (q, ^2^*J* = 31 Hz, C-5), 133.0 (C-4), 122.6 (q, ^1^*J* = 275 Hz, CF_3_), 114.2 (C-3), 113.8 (q, ^3^*J* = 5 Hz, C-6), 112.9 (C-4a), 24.6 (CH_3_). GC-MS (EI, 70 eV) *m/z* 227 (M^+^, 100), 210 (3), 200 (37), 158 (3), 131 (5%).

*2-amino-7-trifluoromethyl-5-methyl-1,8-naphthyridine* (**5b**): Yield 43%.^1^H-NMR (200 MHz, CDCl_3_): *δ* = 8.08 (d, *J =* 9 Hz, 1H, H-4), 7.33 (s, 1H, H-6), 6.90 (d, *J* = 9 Hz, 1H, H-3), 5.98 (s, 2H, NH_2_), 2.67 (s, 3H, CH_3_). ^13^C-NMR (100 MHz, DMSO-*d*_6_): *δ* = 160.3 (C-7), 159.8 (C-8a), 155.2 (C-2), 148.1 (q, ^2^*J* = 34 Hz, C-7), 146.9 (C-5), 132.7 (C-4), 120.8 (q, ^1^*J* = 275 Hz, CF_3_), 114.2 (C-3), 113.8 (q, ^3^*J* = 5 Hz, C-6), 112.9 (C-4a), 17.5 (CH_3_). GC-MS (EI, 70 eV) *m/z*: 227 (M^+^, 100), 210 (3), 200 (37), 158 (3), 131 (5%).

## 4. Conclusions

In summary, we have developed a new, simple and convenient route for the preparation of a new series of 7-alkyl(aryl/heteroaryl)-2-amino-5-trifluoromethyl-1,8-naphthyridines **3a-j**in moderate to good yields, derived from direct cyclocondensation reactions employing acyclic β-alkoxyvinyl trifluoromethyl ketones **1a-j** and 2,6-DAP, under mild conditions by a conventional one-pot procedure. We also developed the synthesis of new 2-amino-5-trifluoromethyl-cycloalka[*b*][1,8]naphthyridines** 3l-m** as fused heteropolycycles from direct or indirect cyclocondensation reactions of 2-trifluoroacetyl-1-methoxycycloalkenes **1l-m** and 2,6-DAP. Furthermore, we have been able to use acyclic and cyclic ketones **1**, for the first time, in the synthesis of trifluoromethylated 2-amino-1,8-naphthyridines, which possess a free amino group for further important derivatizations.
